# Diagnostic value of soluble triggering receptor expressed on myeloid cells in paediatric sepsis: a systematic review

**DOI:** 10.1186/s13052-016-0242-y

**Published:** 2016-04-27

**Authors:** Giuseppe Pontrelli, Franco De Crescenzo, Roberto Buzzetti, Francesca Calò Carducci, Alessandro Jenkner, Donato Amodio, Maia De Luca, Sara Chiurchiù, Elin Haf Davies, Alessandra Simonetti, Elena Ferretti, Martina Della Corte, Luca Gramatica, Susanna Livadiotti, Paolo Rossi

**Affiliations:** Clinical Trial Unit, University Department of Paediatrics, Bambino Gesù Children’s Hospital, IRCCS, Piazza Sant’Onofrio 4, 00100 Rome, Italy; Immunological and Infectious Disease Unit, University Department of Paediatrics, Bambino Gesù Children’s Hospital, IRCCS, Piazza Sant’Onofrio 4, 00100 Rome, Italy; Paediatric European Network for Treatment of AIDS, Via Giustiniani 3, 35128 Padova, Italy

**Keywords:** Triggering receptor expressed on myeloid cells-1, Sepsis, Review, Systematic, Neonates, Children

## Abstract

**Background:**

Differential diagnosis between sepsis and non-infectious inflammatory disorders demands improved biomarkers. Soluble Triggering Receptor Expression on Myeloid cells (sTREM-1) is an activating receptor whose role has been studied throughout the last decade. We performed a systematic review to evaluate the accuracy of plasma sTREM-1 levels in the diagnosis of sepsis in children with Systemic Inflammatory Response Syndrome (SIRS).

**Methods:**

A literature search of PubMed, Cochrane Central Register of Controlled Trials, Cumulative Index to Nursing and Allied Health Literature (CINAHL) and ISI Web of Knowledge databases was performed using specific search terms. Studies were included if they assessed the diagnostic accuracy of plasma sTREM-1 for sepsis in paediatric patients with SIRS. Data on sensitivity, specificity, positive predictive value, negative predictive value, area under receiver operating characteristic curve were extracted. The methodological quality of each study was assessed using a checklist based on the Quality Assessment Tool for Diagnostic Accuracy Studies.

**Results:**

Nine studies comprising 961 patients were included, four of which were in newborns, three in children and two in children with febrile neutropenia. Some data from single studies support a role of sTREM-1 as a diagnostic tool in pediatric sepsis, but cannot be considered conclusive, because a quantitative synthesis was not possible, due to heterogeneity in studies design.

**Conclusions:**

This systematic review suggests that available data are insufficient to support a role for sTREM in the diagnosis and follow-up of paediatric sepsis.

**Electronic supplementary material:**

The online version of this article (doi:10.1186/s13052-016-0242-y) contains supplementary material, which is available to authorized users.

## Background

Sepsis is a syndrome proceeding from Systemic Inflammatory Response Syndrome (SIRS) to invasive infection. A rampant host response can progress to shock and multiple organ failure mediated by immunity, coagulation and intermediate metabolism [1]. Early recognition of sepsis is fundamental in children and adults, since it is still one of the principal causes of death in both populations [2], despite all available antimicrobial and supportive therapies [3].

The pathogenesis of sepsis is complex and not yet fully understood [4]. The first defence against pathogens consists of innate immunity, which prevents dissemination of infection until the adaptive response can occur. While activated, neutrophils and monocytes/macrophages release pro-inflammatory cytokines, attempting to control the infection; an excessive and dysregulated production of cytokines can lead to SIRS and tissue damage. A fine regulation of innate immunity is crucial and several attempts have been made to clarify this complex mechanism [5].

Among several candidate receptors, Triggering Receptor Expressed on Myeloid cells 1 (TREM-1) appears to play a relevant role in the modulation of innate immunity, amplifying or attenuating Toll-Like Receptor (TLR)-induced signals [6, 7]. TREM-1 is a receptor of the immunoglobulin superfamily, expressed on human neutrophils and monocytes [6]. In the early phase of infection, the engagements of Pattern Recognition Receptors (PRRs) by microbial components induce up-regulation of TREM-1. After recognition of a still unknown ligand, TREM-1 associates with a signal transduction molecule called DAP12, triggering the sustained release of pro-inflammatory cytokines (TNF-alpha and IL-1b) and chemokines (IL-8 and monocyte chemotactic protein), which may result in prolonged survival of neutrophils and monocytes at the inflammatory site [8]. TREM-1 activation by itself induces only a modest cellular activation and mediator release, whereas its activation in synergy with TLRs [9] and NOD-like receptors [10] results in a substantial amplification of the immune response. TREM-1 expression was found to be high in the context of inflammatory responses induced by bacterial and fungal infections [11]. More recent data have linked TREM-1 to inflammatory bowel disease, pancreatitis and other non-infectious conditions, calling the idea of a specificity of the TREM-1 pathway into question [12]. Apart from inducing a marked increase in TREM-1 expression, sepsis also induces a soluble form of TREM-1 (sTREM-1), detectable in biological fluids. It is unclear whether sTREM-1 derives from alternative splicing producing secreted receptors isoforms or from the cleavage of extracellular domains of the receptor [6]. However, both cell surface TREM-1 and sTREM-1 are up-regulated during sepsis: due to the undemanding method required to dose the soluble form, this protein has been proposed for the diagnosis of infection in the clinical setting.

Some reviews in adults have investigated the role of TREM-1 in differentiating between infectious and non-infectious conditions. Two previous meta-analyses have found sTREM-1 as a potentially effective biomarker in bacterial infections [13] or bacterial pleural effusions [14]. A recent review examined the role of sTREM-1 in the diagnosis of sepsis in adults [15]. The authors found that sTREM-1 had moderate diagnostic value in differentiating sepsis from SIRS. Specifically, plasma sTREM-1 alone was not considered sufficient for the diagnosis of sepsis in patients with SIRS. Several studies have addressed the role of sTREM-1 in paediatric patient populations. However, these results have never been pooled. Our objective was to systematically review the current level of evidence on sTREM-1 as a diagnostic tool in paediatric sepsis.

## Methods

This work was carried out within the Global Research in Paediatrics (GRiP) framework, an international, EU-funded project aimed at improving the methodology of paediatric research. The authors are involved with the implementation of research methodologies on biomarkers in paediatric sepsis. The study protocol has been agreed within the GRiP framework and has been previously published (http://www.grip-network.org/index.php/cms/en/tools_for_interoperability). This systematic review is in compliance with the PRISMA statement [16].

### Search strategy

A systematic literature search of the PubMed/MEDLINE, Cochrane Library, Cumulative Index to Nursing and Allied Health Literature (CINAHL) and ISI Web of Knowledge databases was carried out, targeting articles assessing sTREM-1 as a diagnostic test for paediatric sepsis. A search algorithm based on a combination of the following terms was used: Systemic Inflammatory Response Syndrome, Bacteraemia, Sepsis, Bacterial infections/diagnosis, Receptors, Immunologic, Biological Markers, Inflammation Mediators, Carrier Proteins/blood, Membrane Glycoproteins/blood, Acute-Phase Proteins, sTREM-1, soluble Triggering Receptor Expressed on Myeloid cells (see Additional file 1: Search strategy).

Publications of regulatory agencies such as the European Medicines Agency (EMA) (scientific guidelines, European public assessment reports of authorized products, opinions of paediatric investigation plans already adopted by the paediatric committee) were examined to describe how the biomarker had been considered for regulatory purposes.

To expand our search, references of the retrieved articles and reviews were hand-searched for additional studies. No lower date limit was set and the search was continued until August 2015. No language limit was used.

### Selection criteria

All studies or subsets of studies on paediatric patients with an assessment of sTREM-1 as a diagnostic test for sepsis were eligible for inclusion.

We excluded articles not within the scope of this review, review articles, editorials or letters, comments, conference proceedings, case reports and studies in patients over 18 years of age. Two groups of authors independently reviewed the articles to assess their eligibility. Disagreements were resolved in a consensus meeting.

### Data extraction

For each study, data concerning the publication (authors, journal, and year of publication), patients and comparisons (gender, age, diagnosis, outcomes, methodology) were collected systematically and independently by two groups of authors.

In order to assess the diagnostic value of sTREM-1 in paediatric sepsis, the following outcomes were extracted: Sensitivity, Specificity, Positive predictive value (PPV), Negative predictive value (NPV) and Area Under Receiver Operating Characteristic (AUC) curve.

### Study quality assessment

Risk of bias in the included studies was assessed independently by two groups of authors using the Quality Assessment Tool for Diagnostic Accuracy Studies (QUADAS) described in the Cochrane Collaboration Handbook as a reference guide. This tool allows rating risk of bias as “low”, “unclear” or “high”. Disagreements were resolved through discussion.

## Results

### Selected studies

**Fig. 1:**
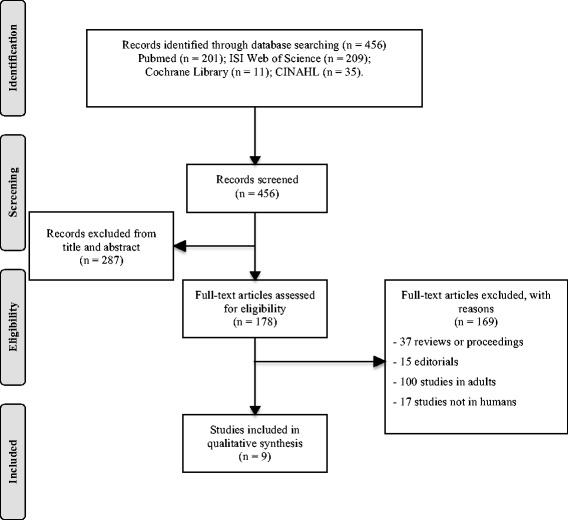
Flow chart. The flow diagram depicts the flow of information through the different phases of this systematic review on the diagnostic value of soluble triggering receptor expressed on myeloid cells in paediatric sepsis

The literature search retrieved 456 records. Following the selection process, nine studies (comprising 961 patients) were identified, four of which had been performed in newborns, three in children and two in children with febrile neutropenia (Fig. 1). Due to heterogeneous study designs, in terms of patient age and use of different comparators, outcome data could not be pooled statistically and are presented qualitatively. Study characteristics are presented in Table 1 and Additional file 2.

**Table 1:** Table of included studies

Study	Number of patients	Gestational Age (Mean + range)	Sex male %	Study Design	Prevalence of infection (%)	Biomarkers	Cut-off	Sens % (95%CI)	Spec % (95%CI)
*Studies in newborns*									
Sarafidis et al. 2010 [17]	52	Infected 35 (24–40) weeks	Infected 57,1%	Prospective	60%	sTREM-1 (ELISA, Quantikine)	143 pg/ml	70 (51-85)	71 (47-88)
					31 infected 22 confirmed sepsis 9 possible sepsis	IL-6	66 pg/ml	80 (61-92)	81 (58-94)
		Non-infected 30 (24–39) weeks	Non-infected 61.2%		21 non infected	sTREM-1/IL-6	144/66	90 (73-98)	62 (38-82)
Schlapbach et al. 2013 [18]	137	Infected 39.9 (34.0–41.6)	Infected 55%	Prospective	24% Infected	sTREM-1 (ELISA *in-house*)	1.25 pg/ml	75	52
						CRP	20 mg/L	36	89
						PCT	2 ng/ml	88	51
						MIF	50 ng/ml	84	62
						PSP	9 ng/ml	79	30
		Non-infected 38.9 (34.0–42.0)	Non-infected 65%		3 proven infection	PSP+PCT		100	28
						>/=1			
					30 probable infections			68	90
						2			
					104 uninfected	PSP+PCT+sTREM			
						>/= 1		100	22
						>/= 2		91	51
						3		55	93
						PSP+PCT+sTREM		100	22
						+CRP			
						>/= 1			
						>/= 2		91	49
						>/= 3		59	86
						4		32	97
Mazzucchelli et al. 2013 [19]	16 patients who developed sepsis among 70 observed patients	Infected 27.5 ± 2.7 weeks	Infected 56.25%	Prospective	22.85%	TREM-1 expression on PMNs by flow cytometer analysis	Absolute levels not significant	56.2%	93.7%
					16 patients with culture proven sepsis		% of expression of TREM on PMN was evaluated 62.12%		
	16 controls chosen among the same 70 observed patients	Controls 26.9 ± 1.0 weeks	Controls 43.75%		16 patient free of infection matched for sex and age	CD64	2.85	87.5%	100%
Adly et al. 2014 [20]	112 septic patients	Culture proven 35+/-3 weeks	Culture proven 53.9%	Prospective	100% (by study definition)	sTREM1 (ELISA, Quantikine)	310 pg/mL	100	100
	40 controls	Culture negative 35.9+/-2,7 weeks	Culture negative 51%		56.3% culture proven		1100 pg/mL (for the prediction of survival)	100	97
		Controls 35.4+/-2 weeks	Controls 55%		43.7% culture negative	CRP	13.5 mg/L	76	72
*Studies in children*									
Chen et al. 2008 [21]	44	Infants with SBI 51.8+/-31.2 day/old	Infants with SBI 69.56%	Prospective	52.27%	sTREM-1 (ELISA, Quantikine)	24.4 pg/ml	87 (78-97)	81 (69-93)
					4.55% bacteraemia				
		Infants without SBI 30.3 +/-30.1 day/old	Infants with SBI 66.6%		4.55% pneumonia				
					4.55% meningitis				
					38.6% Urinary Tract Infections				
Kevan et al. 2011 [22]	24 IF patients	18 months (3 -58 months)	66.6%	Case control	-	sTREM-1 (ELISA, Quantikine)	-	-	-
	Repeated samples (n 65): - IF: 22 - febrile IF without BSI: 10 - IF + BSI: 17 - IF post-tratment:16								
						LBP	-	-	-
	-Control group: 11								
Carrol et al. 2009 [23]	377 patients:	2.3 years, (0.8 -6.1 years)	57%	Prospective	74%	CRP	10 mg/l	100	13
	95 Pneumonia					PCT	0.5 ng/ml	98	27
	282 Meningitidis					sTREM-1 (ELISA, Quantikine)	25 pg/ml	87	15
	190 HIV+					sCD163	5000 ng/ml	66	38
	13 malaria					HMGB1	5 ng/ml	75	40
*Studies in neutropenic children*									
Arzanian et al. 2011 [24]	65	66.2±37 months	53.8%	Prospective	20%	sTREM-1 (ELISA)	525 pg/ml	84.62%	98.08%
Miedema et al. 2011 [25]	29 patients	Bacterial infection 8 years (6-13)	Bacterial infection 36%	Prospective	32.56%	sTREM-1 (ELISA, *in-house*)	-	-	-
						CRP	40 mg/l	t0=69	t0=62
								t24-48 h= 100	t24-48 h= 42
	43 episodes febrile neutropenia	No bacterial infection 8 years (6-12)	No bacterial infection 59%			IL-8	60 ng/l	t0=92	t0=54
						PCT	0.25 ng/ml	t24-48 h= 100	t24-48 h= 57
								t0=79	t0=77
								t24-48 h= 100	t24-48 h= 53

*AUC* Area under curve; *BSI* bloodstream infections; *CI* Confidence interval; *CRP* C-reactive protein; *HMGB1* High-mobility group box 1; *IF* Intestinal failure; *IL-8* Interleukin 8; *LBP* Lipopolysaccharide-Binding Protein; *PSP* Pancreatic stone protein; *SBI* Serious bacterial infection; *sTREM-1* Serum soluble triggering receptor on myeloid cells-1

### Studies in newborns

Sarafidis et al. [17] investigated sTREM-1 for diagnosis of late onset sepsis, finding higher sTREM-1 levels in infected patients, with a sensitivity of 70 % and a specificity of 71 % at a cut-off level of 143 pg/ml. However, IL-6 demonstrated both a higher sensitivity (80 %) and specificity (81 %). It was not clear in this study if the combined use of IL-6 and sTREM-1 would have been more powerful.

Schlapbach et al. [18] found that infected newborns had trend-wise higher sTREM-1 levels (*p* = 0.05), but pancreatic stone protein (PSP), procalcitonin (PCT) and C-reactive protein (CRP) performed better. The sensitivity of sTREM-1 was 75 %, while the specificity was 52 %. The AUC for sTREM-1 was comparable to that of CRP, but PSP and PCT displayed superior performance. Based on ROC curve analyses, sTREM-1 was included in some bioscore models, which were constructed employing two, three or four markers. The bioscore based on PSP and PCT performed similarly to or better than the bioscores including sTREM-1 or CRP, suggesting that neither sTREM-1 nor CRP provided additional independent information. Only PSP and PCT worked as independent predictors of early onset sepsis (EOS) in multivariate logistic regression models.

Mazzucchelli et al. [19] studied both sTREM-1 and TREM-1 expression on polymorphonuclear cells (PMNs) by flow cytometry. The study did not find a statistically significant increase in median sTREM-1 concentration during sepsis. However, a significant reduction in the expression of TREM-1 on PMNs during sepsis was noticed, with a sensitivity of 56.2 % and a specificity of 93.7 %. Data showed that, individually, CD64 was more reliable than TREM-1 expressed on PMNs in identifying newborns with late-onset sepsis, with a sensitivity of 87.5 %, a specificity of 100 % and an AUC of 0.95.

Adly et al. [20] found high sensitivity and specificity for sTREM-1, with an AUC of 1 at a cut-off value of 310 pg/ml. Moreover, they found that sTREM-1, at the cut-off of 1100 pg/ml, was more sensitive than CRP in predicting survival.

### Studies in children

Chen et al. [21] evaluated sTREM-1 in the diagnosis of serious bacterial infection (SBI) in febrile infants less than three months of age. Among SBIs, evidence of bacteraemia was found in 5 % of the population. Most patients suffered from urinary tract infections. In these patients, sTREM-1 at the cut-off level of 24.4 pg/ml showed a superior accuracy to CRP in predicting SBI, with a sensitivity of 87 % and a specificity of 81 % (AUC = 0.88).

Kevan et al. [22] measured the levels of lipopolysaccharide binding protein and sTREM-1 in paediatric patients with intestinal failure and central venous catheter-associated bloodstream infections. This case—control study analysed different infectious episodes in the same patients. As a result, sTREM-1 levels were increased during all infectious episodes but not specifically bacteraemic episodes.

Carrol et al. [23] studied the accuracy of sTREM-1 and four additional biomarkers in diagnosing sepsis in children presenting with severe infection and high prevalence of malaria and HIV. In this case, sTREM-1 was not superior to CRP and PCT, exhibiting high sensitivity (87 %), but low specificity (15 %).

### Studies in children with febrile neutropenia

Miedema et al. [24] studied sTREM-1 together with CRP, IL-8 and PCT in childhood cancer patients with febrile neutropenia. The aim of this study was to prove the diagnostic value of biomarkers in detecting bacterial infections. sTREM-1 was below the detection limit (likely because both monocyte and neutrophil counts were low by definition), and therefore appeared unsuitable as biomarker in febrile neutropenia.

Arzanian et al. [25] demonstrated a significant association between sTREM-1 levels and the presence of bacteraemia and fungaemia in febrile neutropenic patients with cancer. They used a cut-off point of 525 pg/ml for sTREM-1 as detected by ELISA. In a population of 65 patients (mean age of five years), they found high sensitivity (85 %) and specificity (98 %) for sTREM-1 in the detection of blood infections (AUC = 0.96).

### Quality assessment

Assessment of methodological quality of included articles according to the QUADAS criteria is reported in Table 2. Six studies presented spectrum bias. This is the bias originating from the selection of a population that certainly harbours the disease, which is compared to a population of unaffected subjects. Under spectrum bias both sensitivity and specificity are artificially increased, suggesting an overrated diagnostic accuracy. All studies were unclear as to whether the reference standard had been interpreted without prior knowledge of the index test. Overall, the reference standard had been correctly defined both in newborns and in children. Selection criteria comprising inclusion and exclusion criteria of each study are reported in Table 3. We decided to rate a bias in the QUADAS question for the selection criteria in all studies that did not clearly report the exclusion criteria employed.

**Table 2:** Quality assessment (QUADAS)

	*Chen* et al. *2008* [21]	*Carrol* et al. *2009* [23]	*Sarafidis* et al. *2010* [17]	*Kevan* et al. *2011* [22]	*Miedema* et al. *2011* [25]	*Arzanian* et al. *2011* [24]	*Schlapbach* et al. *2013* [18]	*Mazzucchelli* et al. *2013* [19]	*Adly* et al. *2014* [20]
Was the spectrum of patients representative of the patients who will receive the test in practice?	−	−	+	−	−	+	+	−	−
Were selection criteria clearly described?	−	+	+	+	−	+	−	−	+
Is the reference standard likely to correctly classify the target condition?	+	+	+	+	+	+	+	+	+
Is the time period between reference standard and index test short enough to be reasonably sure that the target condition did not change between the two tests?	+	+	+	+	+	+	+	+	+
Did the whole sample or a random selection of the sample, receive verification using a reference standard of diagnosis?	+	+	+	+	?	+	−	+	+
Did patients receive the same reference standard regardless of the index test result?	+	+	+	+	+	+	+	+	+
Was the reference standard independent of the index test (i.e. the index test did not form part of the reference standard)?	+	+	+	+	+	+	+	+	+
Was the execution of the index test described in sufficient detail to permit replication of the test?	+	+	+	+	−	+	+	+	+
Was the execution of the reference standard described in sufficient detail to permit its replication?	+	+	+	+	−	+	+	+	+
Were the index test results interpreted without knowledge of the results of the reference standard?	+	+	+	+	+	+	+	+	+
Were the reference standard results interpreted without knowledge of the results of the index test?	?	?	?	?	?	?	?	?	?
Were the same clinical data available when test results were interpreted as would be available when the test is used in practice?	+	+	+	+	+	+	+	+	+
Were uninterpretable/intermediate test results reported?	+	+	+	+	+	?	+	?	?
Were withdrawals from the study explained?	+	+	+	+	+	?	+	?	?

“+” = low risk of bias; “−” = high risk of bias; “?” = unclear risk of bias

**Table 3:** Selection criteria of the single studies

	*Inclusion criteria*	*Exclusion criteria*
*Chen* et al. *2008*	Febrile infants < 3 months with suspected serious bacterial infection (SBI): UTI, pneumonia, positive CSF or blood colture.	Not disclosed.
*Carrol* et al. *2009*	Children with suspected Serious Bacterial Infection (meningitides or Pneumonia) of a Malawian Hospital. Age: 2 months–16 years.	Significant co-existing morbidity.
*Sarafidis* et al. *2010*	Admission to a NICU of a single university hospital for suspected Late Onset Sepsis (LOS) >3 days.	Mothers with clinical chorioamnionitis; early-onset sepsis; congenital infections or anomalies.
*Kevan* et al. *2011*	Pediatric intestinal failure (IF) patients with central venous catheter (CVC) of a children hospital. Age: between 3 months and 4 years.	Small bowel, liver/small bowel, or multivisceral transplant; known underlying immune disorder; current diagnosis of infection other than CVC-BSI; immune suppressant medications or systemic antibiotics for more than 24 hours before inclusion.
*Miedema* et al. *2011*	Febrile neutropenia and oncological underlying disease.	Not disclosed.
*Arzanian* et al. *2011*	Fever >38 °C for at least one hour or >38,3° in a single measurement; absolute neutrophil count of less than 500/mm3.	Patients under treatment with GCSF; patients already on antibiotics before the beginning of fever and neutropenia except for prophylaxis.
*Schlapbach* et al. *2013*	Neonates >34 weeks admitted within the first 72 h with suspicion of sepsis (Early onset sepsis).	Not disclosed.
*Mazzucchelli* et al. *2013*	Gestational age younger than 32 weeks and/or birth weigh less than 1500 g free of infection.	Not disclosed.
*Adly* et al. *2014*	Newborns with clinical and laboratory signs of sepsis.	Congenital infections; malformations; major chromosomal abnormalities; prior use of intravenous immunoglobulins.

## Discussion

The results of our review show preliminary evidence for a role of sTREM-1 in the diagnostic workup of sepsis in newborns and children. It was not possible to obtain quantitative results due to the small number of studies, which included heterogeneous populations. We divided the retrieved studies in three diagnostic categories: studies in newborns, in children and in children with febrile neutropenia.

### Studies in newborns

The four newborn studies were affected by variability concerning inclusion criteria: Sarafidis et al. [17] included late onset sepsis (LOS), Schlapbach et al. [18] early onset sepsis (EOS), Adly et al. [20] and Mazzucchelli et al. [19] included both. Concerning age, a variable mix of term and preterm babies was included in three studies, whereas in the study by Mazzucchelli et al. [19] only preterm babies were included. Two articles clearly disclosed the exclusion criteria: Adly et al. [20] and Sarafidis et al. [17] both left out congenital abnormalities or congenital infections, Sarafidis et al. [17] excluded babies born from mothers with chorioamnionitis and Adly et al. [20] excluded babies previously treated with intravenous immunoglobulin. In three studies, sTREM-1 plasma concentration was evaluated by ELISA. In the study by Mazzucchelli et al. [19], sTREM-1 dosage correlated weakly with the diagnosis of sepsis, whereas a strong correlation was found for the cytofluorimetric evaluation of TREM-1 membrane expression on neutrophils and monocytes. Relevant differences were found in terms of methodology: two out of four studies [19, 20] were affected by spectrum bias. This was clearly mirrored by the very high AUC: 80 % in Mazzucchelli et al. [19] and even 100 % in Adly et al. [20], much higher than the AUC observed in the other two studies: 73 % for Sarafidis et al. [17] and 62 % for Schlapbach et al. [18]. Actually, in Schlapbach et al. [18], sTREM-1 accuracy was comparable to that of CRP and PSP (62 % vs 66 % and 69 %, respectively), but was much lower than PCT (77 %). sTREM-1 might have been more accurate in EOS than in LOS, but a well-designed study would be needed; the evaluation of a biomarker combination (such as PCT + TREM) could also be of interest in order to assess a possible gain in accuracy and cost-effectiveness.

### Studies in children

The three studies in children were characterized by a high variability among clinical conditions. Chen et al. [21] included infants less than three months of age with suspected serious bacterial infection, including urinary tract infections, pneumonia, positive blood or cerebrospinal fluid culture, reporting a higher accuracy of sTREM-1 (AUC 88 %) as compared to CRP or the immature-to-total neutrophil ratio. Kevan et al. [22] included a population of paediatric intestinal failure patients with central venous catheters and evaluated the role of sTREM-1 in device-associated bloodstream infections: in this setting, sTREM-1 showed poor discriminating power for bacteraemia (AUC 57 %), probably because of the strong confounding factor of the impaired intestinal wall. The last study [23] was carried out in a developing country, thus being subject to the peculiar epidemiological features of such context, primarily a high prevalence of HIV, which in fact affected about 50 % of the patients. sTREM-1 performed no better than CRP (AUC 50 % vs. 52 %) and far worse than PCT (81 %) in this environment.

### Studies in children with febrile neutropenia

Two studies were performed in children with febrile neutropenia. In these studies SIRS was not employed as an inclusion criterion, because chemotherapy-induced neutropenia represents *per se* a condition of increased susceptibility to infections, requiring prompt clinical evaluation. The two studies showed different results: in Arzanian et al. [24], a very high cut-off value for sTREM-1 was used, resulting in a high accuracy in diagnosing bacteraemia and fungaemia in febrile neutropenic patients, whereas in Miedema et al. [25], sTREM-1 accuracy could not be evaluated since sTREM-1 appeared to be undetectable at presentation in most patients.

### Limitations

Like other systematic reviews of diagnostic tests, our work has some limitations. Firstly, The included studies opted for very different sTREM-1 cut-off values and measuring techniques (Table 1 and Additional file 2); Secondly, publication bias (lower probability of publication of negative results) is known to be more difficult to avoid in observational studies of diagnostic tests than in randomized controlled trials [26]; Thirdly, in several studies blinding of reference standard results and the ‘blindness’ of study design were not fully reported; Fourthly, no article reported if the reference standard results had been interpreted without prior knowledge of the results of the index test; Fifthly, several studies were affected by spectrum bias; Sixthly, given that different methodologies were used, it was not possible to perform a meta-analysis, nor were we able to obtain a pooled estimate of accuracy for sTREM-1.

## Conclusions

A specific marker for the early detection of paediatric sepsis would be highly desirable. Reviewed data support a role of sTREM-1 as a diagnostic tool in this setting, but cannot be considered conclusive. Some evidences suggest that the determination of sTREM-1 in combination with other biomarkers could achieve a better performance than each biomarker alone. Indeed, it would be very important to standardize measuring techniques in order to achieve more robust and comparable results. Recent diagnostic developments, such as multiplex bead array assays, offer promising opportunities [27].

We believe that large, prospective studies exploring the role of sTREM-1would be necessary to overcome heterogeneity and inconsistent results.

We recommend to:

1. Harmonize methodology (using agreed case definitions of suspected and confirmed sepsis, Early and Late Onset Sepsis);
2. Investigate combination of sTREM-1 with other biomarkers, such as CRP, PCT, TNF-alpha or IL-6, and a scoring system bridging clinical and laboratory findings.

At present, sTREM-1 should be considered an interesting exploratory biomarker for paediatric sepsis.

## Additional file

Additional file 1: Supplemental Material—Search strategy. (DOC 34 kb) Additional file 2: Table S1. (DOCX 48 kb)
