# Supplementary material for: Diagnostic value of soluble triggering receptor expressed on myeloid cells in paediatric sepsis: a systematic review
Source: Ital J Pediatr. 2016 Apr 27;42:44. doi: 10.1186/s13052-016-0242-y (PMC4847353; doi:10.1186/s13052-016-0242-y)
Supplement: Additional file 1: — Supplemental Material—Search strategy. (DOC 34 kb) [file 13052_2016_242_MOESM1_ESM.doc]

**Supplementary material 1**. Search strategy.

Search strategy: Medline/Pubmed

1. exp "Systemic Inflammatory Response Syndrome"[Mesh]/
2. Bacteremia [Mesh].mp
3. Sepsis[Mesh].mp
4. “Bacterial infections/diagnosis"[Mesh].mp
5. 1 OR 2 OR 3 OR 4
6. “Receptors, Immunologic"[Mesh].mp
7. "Biological Markers"[Mesh].mp
8. Inflammation Mediators"[Mesh].mp
9. "Carrier Proteins/blood"[Mesh].mp
10. "Membrane Glycoproteins/blood"[Mesh].mp
11. “Acute-Phase Proteins"[Mesh].mp
12. 6 OR 7 OR 8 OR 9 OR 10 OR 11
13. “sTREM-1”.mp
14. “soluble Triggering Receptor Expressed on Myeloid cells”.mp
15. "TREM1 protein, human".mp
16. OR trem.mp
17. OR strem.mp
18. OR s-trem.mp
19. OR "Triggering Receptor Expression on Myeloid cells".mp
20. OR trem?1 .mp
21. 13 OR 14 OR 15 OR 16 OR 17 OR 18 OR 19 OR 20
22. 5 AND 12 AND 21

Search strategy: CINHAL

1. (MM “Systemic Inflammatory Response Syndrome+”)
2. “bacteremia”
3. “sepsis”
4. “bacterial infections”
5. “sirs”
6. 1 OR 2 OR 3 OR 4 OR 5
7. “TREM1 protein human"
8. “trem”
9. “strem”
10. “s-trem”
11. "Triggering Receptor Expression on Myeloid cells"
12. 7 OR 8 OR 9 OR 10
13. 6 AND 12

Search strategy: Isi Web of Science

1. “Systemic Inflammatory Response Syndrome”.mp
2. “bacteremia”.mp
3. “sepsis”.mp
4. “bacterial infections”.mp
5. “sirs”.mp
6. 1 OR 2 OR 3 OR 4 OR 5
7. TREM1 protein human" .mp
8. “trem” .mp
9. “strem” .mp
10. “s-trem” .mp
11. "Triggering Receptor Expression on Myeloid cells" .mp
12. 7 OR 8 OR 9 OR 10 OR 11
13. 6 AND 12

Search strategy: Cochrane Library

1. “Systemic Inflammatory Response Syndrome”
2. bacteremia
3. sepsis
4. “bacterial infections”
5. sirs
6. 1 OR 2 OR 3 OR 4 OR 5
7. “TREM1 protein”
8. trem
9. strem
10. s-trem
11. "Triggering Receptor Expression on Myeloid cells"
12. 7 OR 8 OR 9 OR 10 OR 11
13. 6 AND 12
