# Supplementary material for: Diagnostic value of soluble triggering receptor expressed on myeloid cells in paediatric sepsis: a systematic review
Source: Ital J Pediatr. 2016 Apr 27;42:44. doi: 10.1186/s13052-016-0242-y (PMC4847353; doi:10.1186/s13052-016-0242-y)
Supplement: Additional file 2: — Table S1. (DOCX 48 kb) [file 13052_2016_242_MOESM2_ESM.docx]

| **Study** | **Number of patients** | **Gestational Age (Mean + range)** | **Sex**  **male %** | **Study**  **Design** | **Prevalence**  **of infection**  **(%)** | **Biomarkers** | **Cut-off** | **Sens % (95%CI)** | **Spec %**  **(95%CI)** | **PPV %**  **(95%CI)** | **NPV % ­­(95%CI)** | **AUC**  **(95% CI)** | **LR+**  **(95%CI)** | **LR-**  **(95%CI)** |
| --- | --- | --- | --- | --- | --- | --- | --- | --- | --- | --- | --- | --- | --- | --- |
| *Studies in newborns* | | | | | | | | | | | | | | |
| *Sarafidis et al. 2010*  *[17]* | 52 | Infected  35 (24–40) weeks  Non-infected  30 (24–39) weeks | Infected 57,1%  Non-infected 61.2% | Prospective | 60%  31 infected  22 confirmed sepsis  9 possible sepsis  21 non infected | sTREM-1  (ELISA, Quantikine)  IL-6  sTREM-1/IL-6 | 143 pg/ml  66 pg/ml  144/66 | 70  (51-85)  80  (61-92)  90  (73-98) | 71  (47-88)  81  (58-94)  62  (38-82) | 78  (59-91)  86  (67-95)  77  (59-89) | 62  (41-81)  74  (59-89)  81  (54-96) | 0.73  (0.59-0.88)  0.89  (0.81-0.98)  / | 2.4  4.2  2.4 | - |
| *Schlapbach et al. 2013 [18]* | 137 | Infected  39.9 (34.0–41.6)  Non-infected  38.9 (34.0–42.0) | Infected  55%  Non-infected  65% | Prospective | 24%  Infected  3 proven infection  30 probable infections  104 uninfected | sTREM-1 (ELISA *in-house*)  CRP  PCT  MIF  PSP | 1.25 pg/ml  20 mg/L  2 ng/ml  50 ng/ml  9 ng/ml | 75  36  88  84  79 | 52  89  51  62  30 | 34  52  39  29  39 | 87  82  92  85  90 | 0.62 (0.51-0.73)  0.66 (0.55-0.77)  0.77 (0.66-0.87)  0.54 (0.41-0.67)  0.69 (0.59-0.80) | 1.6  3.4  1.8  1.2  2.1 | 0.48  0.71  0.24  0.54  0.35 |
|  |  |  |  |  |  | PSP+PCT  >/=1  2  PSP+PCT+sTREM  >/= 1  >/= 2  3  PSP+PCT+sTREM  +CRP  >/= 1  >/= 2  >/= 3  4 |  | 100  68  100  91  55  100  91  59  32 | 28  90  22  51  93  22  49  86  97 | 33  71  32  41  75  32  40  62  78 | 100  89  100  94  85  100  94  85  79 | 0.83  (0.74–0.93)  0.82  (0.72–0.92)  0.81  (0.71–0.91) | 1.4  6.7  1.3  1.9  8  1.3  1.8  4.4  9.4 | 0  0.36  0  0.18  0.49  0  0.19  0.47  0.71 |
| *Mazzucchelli et al. 2013*  [19] | 16 patients who developed sepsis among 70 observed patients  16 controls chosen among the same 70 observed patients | Infected  27.5 ± 2.7 weeks  Controls  26.9 ± 1.0 weeks | Infected  56.25%  Controls  43.75% | Prospective | 22.85%  16 patients with culture proven sepsis  16 patient free of infection matched for sex and age | TREM-1 expression on  PMNs by flow  cytometer analysis  CD64 | Absolute levels not significant  % of expression of TREM on PMN was evaluated  62.12%  2.85 | 56.2%  87.5% | 93.7%  100% | - | - | 0.8  0.95 | - | - |
| *Adly et al. 2014*  [20] | 112 septic patients  40 controls | Culture proven  35+/-3 weeks  Culture negative  35.9+/-2,7 weeks  Controls  35.4+/-2 weeks | Culture proven  53.9%  Culture negative  51%  Controls  55% | Prospective | 100%  (by study definition)  56.3% culture proven  43.7% culture negative | sTREM1 (ELISA,  Quantikine)  CRP | 310 pg/mL  1100 pg/mL (for the prediction of survival)  13.5 mg/L | 100  100  76 | 100  97  72 | - | - | 1  (0.696-1.015)  0.762  (0.612–0.925) | - | - |

**Table 1.** Table of included studies.

| **Study** | **Number of patients** | **Age (Mean + range)** | **Sex**  **male %** | **Study**  **Design** | **Prevalence**  **of infection**  **(%)** | **Biomarkers** | **Cut-off** | **Sens % (95%CI)** | **Spec %**  **(95%CI)** | **PPV %**  **(95%CI)** | **NPV % (95%CI)** | **AUC**  **(95% CI)** | **LR+**  **(95%CI)** | **LR-**  **(95%CI)** |
| --- | --- | --- | --- | --- | --- | --- | --- | --- | --- | --- | --- | --- | --- | --- |
| *Studies in children* | | | | | | | | | | | | | | |
| *Chen et al. 2008*  [21] | 44 | Infants with SBI  51.8+/-31.2 day/old  Infants without SBI  30.3 +/-30.1 day/old | Infants with SBI  69.56%  Infants with SBI  66.6% | Prospective | 52.27%  4.55% bacteraemia  4.55%  pneumonia  4.55%  meningitis  38.6%  Urinary Tract Infections | sTREM-1  (ELISA, Quantikine) | 24.4 pg/ml | 87  (78-97) | 81  (69-93) | 83  (64-93) | 85  (64-95) | 0.88  (0.78-0.99) | 4.6  (1.9-11.2) | 0.2  (0.1-0.4) |
| *Kevan et al. 2011*  [22] | 24 IF patients  Repeated samples (n 65):  - IF: 22  - febrile IF without BSI: 10  - IF + BSI: 17  - IF post-tratment:16  -Control group: 11 | 18 months  (3 -58 months) | 66.6% | Case  control | - | sTREM-1  (ELISA, Quantikine) | - | - | - | - | - | 0.57 | - |  |
|  |  |  |  |  |  | LBP | - | - | - | - | - | 0.82 | - |  |
| *Carrol et al. 2009*  *[23]* | 377 patients:  95 Pneumonia  282 Meningitidis  190 HIV+  13 malaria | 2.3 years,  (0.8 -6.1 years) | 57% | Prospective | 74% | CRP | 10 mg/l | 100 | 13 | 77 | 100 | 0.52 (0.43-0.61) | 1,1 | 0,0 |
|  |  |  |  |  |  | PCT | 0.5 ng/ml | 98 | 27 | 79 | 80 | 0.81 (0.73-0.89) | 1,3 | 0,1 |
|  |  |  |  |  |  | sTREM-1  (ELISA, Quantikine) | 25 pg/ml | 87 | 15 | 74 | 29 | 0.50 (0.41-0.60) | 1,0 | 0,9 |
|  |  |  |  |  |  | sCD163 | 5000 ng/ml | 66 | 38 | 75 | 27 | 0.86 (0.79-0.92) | 1,1 | 0,9 |
|  |  |  |  |  |  | HMGB1 | 5 ng/ml | 75 | 40 | 77 | 37 | 0.59 (0.50-0.69) | 1,3 | 0,6 |
| *Studies in neutropenic children* | | | | | | | | | | | | | | |
| *Arzanian et al. 2011*  [24] | 65 | 66.2±37  months | 53.8% | Prospective | 20% | sTREM-1  (ELISA) | 525 pg/ml | 84.62% | 98.08% | 91.67% | 96.23% | 0.96 | ∞ | 0.15 |
| *Miedema et al. 2011*  *[25]* | 29 patients  43 episodes  febrile neutropenia | Bacterial infection  8 years (6-13)  No bacterial infection  8 years (6-12) | Bacterial infection  36%  No bacterial infection  59% | Prospective | 32.56% | sTREM-1  (ELISA, *in-house*) | - | - | - | - | - | - | - | - |
|  |  |  |  |  |  | CRP | 40 mg/l | t0=69  t24-48 h= 100 | t0=62  t24-48 h= 42 | - | - | 0.61 (0.39-0.80) | - | - |
|  |  |  |  |  |  | IL-8 | 60 ng/l | t0=92  t24-48 h= 100 | t0=54  t24-48 h= 57 | - | - | 0.81 (0.66-0.97) | - | - |
|  |  |  |  |  |  | PCT | 0.25 ng/ml | t0=79  t24-48 h= 100 | t0=77  t24-48 h= 53 | - | - | 0.77 (0.61-0.93) | - | - |

Notes: AUC = Area under curve; BSI = bloodstream infections; CI = Confidence interval; CRP = C-reactive protein; HMGB1 = High-mobility group box 1; IF = Intestinal failure; IL-8 = Interleukin 8; LBP = Lipopolysaccharide-Binding Protein; LR+ = Positive likelihood ratio; LR- = Negative likelihood ratio; MIF = Macrophage migration inhibitory factor; NPV = Negative predictive value; PPV = Positive predictive value; PSP = Pancreatic stone protein; SBI = Serious bacterial infection; sTREM-1 = Serum soluble triggering receptor on myeloid cells-1.
